# Treatment-Resistant Fascicular Ventricular Tachycardia: A Case Report

**DOI:** 10.7759/cureus.102014

**Published:** 2026-01-21

**Authors:** Ahmed Abotabekh, Mahmoud Eldesouky, Safiyyah Suleman, Riyaz Somani

**Affiliations:** 1 Cardiology, University Hospitals of Leicester NHS Trust, Leicester, GBR; 2 Cardiovascular Sciences, Glenfield Hospital, Leicester, GBR

**Keywords:** fascicular ventricular tachycardia, life-threatening arrhythmia, ventricular arrhythmia, verapamil-sensitive ventricular tachycardia, vt ablation

## Abstract

Fascicular ventricular tachycardia (VT) is a form of VT that arises through a re-entrant pathway utilising the Purkinje fibres. It can be categorised based on the anatomical origin of the re-entrant pathway. Fascicular VT typically affects young males. We present a case of a young male patient with treatment-resistant fascicular VT. A young male in his twenties spent much of his adolescence (8 years) suffering from sudden-onset and offset ‘panic attack’-like symptoms. A routine preoperative ECG showed posterior fascicular VT. After a poor response to medical therapy, the patient underwent two catheter ablation procedures, with a final good symptomatic outcome. Fascicular VT is often difficult to diagnose and can be misinterpreted as supraventricular tachycardia (SVT) given the narrow QRS. The mainstay of medical management is verapamil; however, more invasive treatments such as catheter ablation are available, yielding high success rates.

## Introduction

Fascicular ventricular tachycardia (VT) is an unusual type of VT, first recorded in 1979 by Belhassen B, et al. [1,2]. Fascicular VT arises from a re-entry circuit utilising the Purkinje fibres [1-3]. Depending on which fascicle of the Purkinje fibres is affected, the ECG appearance varies. The common feature of the different forms of fascicular VT is that there is a relatively narrower QRS complex in comparison to other types of VT [4,5].

## Case presentation

**Figure 1:**
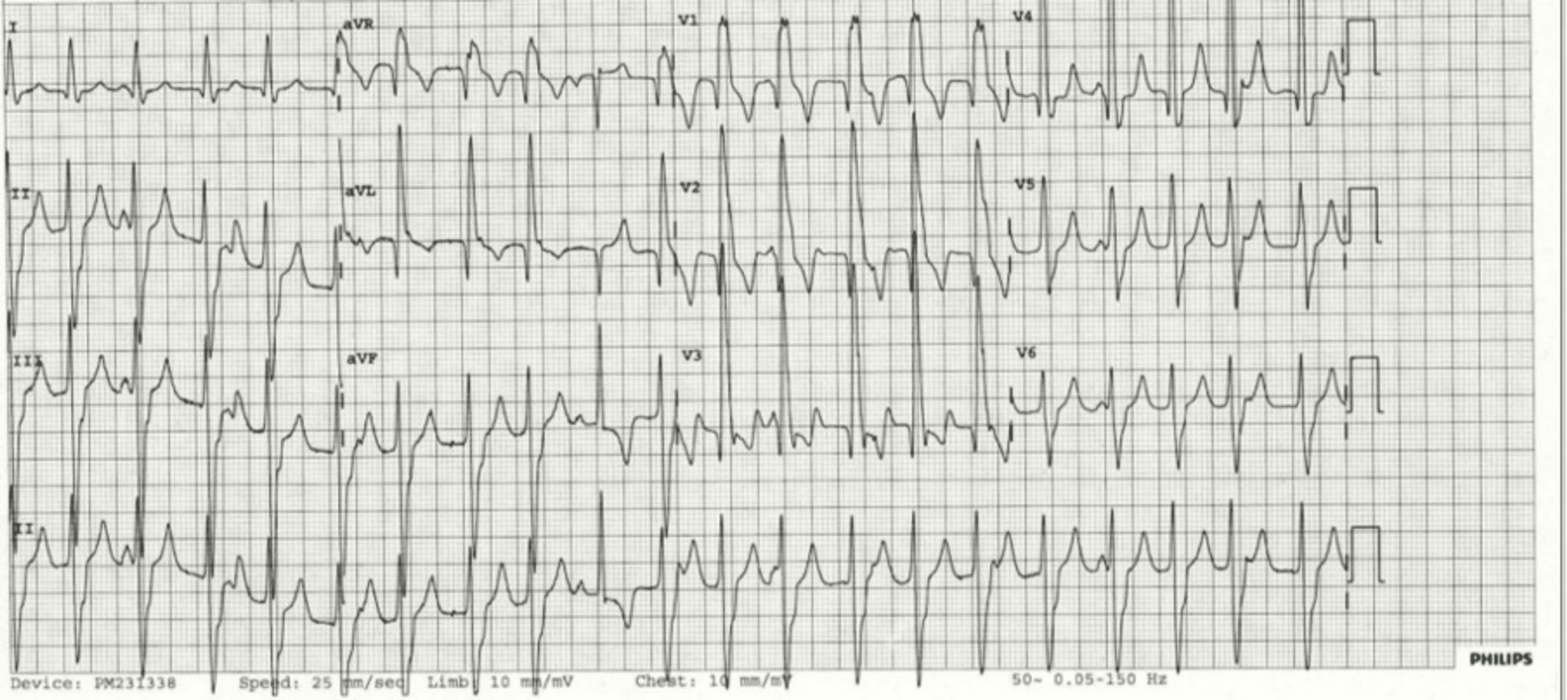
12-lead ECG showing a QRS duration of approximately 130 ms, left axis deviation, and a right bundle branch block morphology, consistent with posterior fascicular ventricular tachycardia.

A young male in his mid-twenties was admitted to hospital for a surgical procedure and was found to have fascicular VT on a routine ECG. He reported no relevant past medical history and was awaiting surgery for a sports-related knee injury. The patient had never smoked or consumed alcohol and denied any recreational drug use. There was no reported family history of cardiac disease. On initial review by the cardiology team, the patient did not report any significant symptoms. However, on further detailed questioning, he reported episodes of palpitations, labelled as ‘panic attacks’. He described short-lived episodes where his heart started beating very fast and he felt lightheaded. In retrospect, these ‘panic attacks’ could have been manifestations of previous episodes of fascicular VT. His presenting ECG, in keeping with fascicular VT, can be seen in Figure 1.

Following this incidental finding, the patient underwent a cardiac MRI stress perfusion scan to rule out any underlying cardiomyopathy. The scan showed no evidence of regional wall motion abnormalities, infarction, scarring, or fibrosis, and there was no inducible ischaemia. There was global hypokinesis of the left ventricle with a left ventricular ejection fraction (LVEF) of 45%. The reduced ejection fraction was assessed to be likely related to tachycardia-induced cardiomyopathy, secondary to sustained VT.

The patient underwent a catheter ablation attempt, during which a linear lesion set was delivered transecting the posterior fascicle, reverting him to sinus rhythm. Unfortunately, a few weeks post-ablation, the patient noted chest pain and recurrent palpitations. When reviewed in clinic, his ECG confirmed recurrence of VT with the same morphology as the initial VT.

The patient was admitted from clinic and, on this occasion, was treated medically with IV verapamil, which reverted him to sinus rhythm. He was then discharged on oral verapamil before returning for a second catheter ablation procedure, once again targeting the posterior fascicle. Since this second procedure (14 months ago at the time of writing), he has been free of recurrent VT, suggesting a successful outcome. Repeat cardiac imaging is planned, with the hope that left ventricular function will have normalised following treatment of the tachycardia.

## Discussion

Fascicular VT is a condition widely believed to be associated with a re-entrant circuit occurring as a result of abnormal Purkinje fibres. These fibres rely on slow calcium conduction, allowing re-entry to form. This pathophysiology, particularly the dependence on calcium channels, is what makes calcium channel blockers the mainstay of medical treatment for this condition [3].

Fascicular VT tends to occur in younger adults, typically aged 14-40 years, the majority of whom are men [6]. Patients typically present with a history of episodic dizziness, palpitations, and occasionally syncope. These episodes tend to occur at rest; however, there might be notable triggers such as stress, excitement, exercise, and/or infection [4].

**Figure 2:**
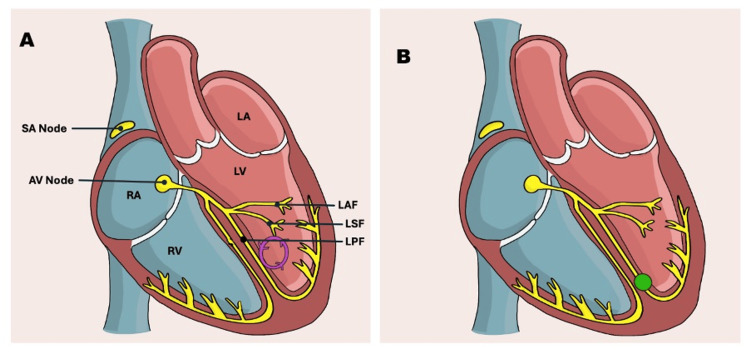
Original schematic design of the fascicular re-entrant circuit (A) and the ablation lesion target (B). A: Schematic diagram of the fascicular re-entrant circuit. B: Schematic diagram of the ablation lesion target. SA: Sinoatrial; AV: Atrioventricular; RA: Right atrium; LA: Left atrium; RV: Right ventricle; LV: Left ventricle; LAF: Left anterior fascicle; LSF: Left septal fascicle; LPF: Left posterior fascicle. Schematic diagram created by the authors.

There are three types of fascicular VT, classified based on the anatomical origin of the re-entrant pathway [4,5]. The first and most common type is posterior fascicular VT, which accounts for the majority of cases (90-95%) and typically arises near the left posterior fascicle, as seen in Figure 2 [4,5]. Posterior fascicular VT presents with right bundle branch block, left axis deviation, and a QRS duration between 100 ms and 140 ms [4,5]. The second type is anterior fascicular VT, which makes up 5-10% of cases. Anterior fascicular VT arises near the left anterior fascicle, presenting with right bundle branch block, right axis deviation, and a QRS duration between 100 ms and 140 ms [4,5]. Finally, the third and rarest type of fascicular VT, often termed upper septal fascicular VT, arises from the upper septum region [4,5]. In upper septal fascicular VT, ECG changes can be variable, and patients might present with either right bundle branch block or left bundle branch block, a normal axis, and a QRS duration usually <120 ms [4,5].

Fascicular VT is frequently misdiagnosed as other tachyarrhythmias, including supraventricular tachycardia with aberrant conduction, outflow tract VT, and scar-related VT. Table 1 summarises the key ECG features that help distinguish these entities, although overlap exists and electrocardiographic findings may vary across subtypes [4-16].

**Table 1:** Typical electrocardiographic findings in fascicular ventricular tachycardia compared with various tachyarrhythmias. LV: Left ventricle; AV: Atrioventricular; RBBB: Right bundle branch block; BBB: Bundle branch block; LBBB: Left bundle branch block; LAD: Left axis deviation; RAD: Right axis deviation.

Feature	Fascicular ventricular tachycardia	Supraventricular tachycardia with aberrancy	Right ventricular outflow tract ventricular tachycardia	Left ventricular outflow tract ventricular tachycardia	Scar-related ventricular tachycardia
Origin	LV fascicles	Atria/AV node	Right ventricular outflow tract	Left ventricular outflow tract and aortic root region	Macro-reentry around scar anywhere in the ventricular myocardium
QRS duration	110–140 ms	≥120 ms	Septal origin <140 ms; free-wall origin ≥140 ms	Typically >120 ms; variable by site	Often wide; varies depending on scar location
QRS morphology	RBBB pattern	Any BBB pattern	Typically LBBB with inferior axis	Typically RBBB, but can resemble LBBB depending on exact origin	Variable depending on chamber and exit site
Axis	LAD (posterior) or RAD (anterior)	Normal/non-extreme	Inferior axis	Often inferior ± rightward; varies by subtype	Varies depending on scar location
AV dissociation	May be present	Absent	May be present	May be present	May be present
Capture/fusion	Rare	Absent	May be present	May be present	May be present
Precordial concordance	Uncommon	Absent	Uncommon	Uncommon	May be present

The main medical treatment for fascicular VT is verapamil, which is often effective acutely, although it may be less effective as a long-term strategy, as was the case here. Verapamil should ideally only be used in stable patients with an established diagnosis. While some case reports have shown success in treating fascicular VT with adenosine, it is usually unresponsive to this medication. Catheter ablation for fascicular VT has a success rate of 70%-90%, although there is a risk of recurrence of between 5% and 12.5% [5]. It is important to note, however, that left ventricular catheter ablation, regardless of indication, does come with risks, including groin complications (e.g. haematoma, false aneurysm), iatrogenic left bundle branch block, or AV block [6]. Rarer complications include cardiac perforation with associated tamponade, thrombus formation leading to stroke, and potential traumatic damage to the mitral or aortic valves caused by catheter manipulation [6]. The target site for catheter ablation in posterior fascicular VT can be seen as a green dot in picture B in Figure 2.

## Conclusions

Fascicular VT is an arrhythmia that occurs due to abnormal Purkinje fibres, which have slow calcium conduction, allowing a re-entry pathway to form. Fascicular VT typically affects young males aged 14-40 years and can commonly be misdiagnosed, which is why it is vital for clinicians to obtain an ECG at the onset of symptoms to aid diagnosis. Fascicular VT is often difficult to diagnose and can be misinterpreted as supraventricular tachycardia (SVT) given the relatively narrow QRS. The mainstay of medical management is verapamil; however, more invasive treatments such as catheter ablation are available, yielding high success rates.
